# Characteristics of hospitalized patients with SARS-CoV-2 infection during successive waves of the COVID-19 pandemic in a reference hospital in Spain

**DOI:** 10.1038/s41598-022-22145-9

**Published:** 2022-10-17

**Authors:** Simona Iftimie, Ana F. López-Azcona, Maria José Lozano-Olmo, Anna Hernández-Aguilera, Salvador Sarrà-Moretó, Jorge Joven, Jordi Camps, Antoni Castro

**Affiliations:** 1 Department of Internal Medicine, Hospital Universitari de Sant Joan, Institut d’Investigació Sanitària Pere Virgili, Universitat Rovira i Virgili, Av. Dr. Josep Laporte 2, 43204 Reus, Spain; 2 Department of Pathology, Hospital Universitari de Sant Joan, Institut d’Investigació Sanitària Pere Virgili, Universitat Rovira I Virgili, Av. Dr. Josep Laporte 2, 43204 Reus, Spain; 3 Biomedical Research Unit, Hospital Universitari de Sant Joan, Institut d’Investigació Sanitària Pere Virgili, Universitat Rovira I Virgili, Av. Dr. Josep Laporte 2, 43204 Reus, Spain; 4 Department of Emergencies, Hospital Universitari de Sant Joan, Institut d’Investigació Sanitària Pere Virgili, Universitat Rovira I Virgili, Av. Dr. Josep Laporte 2, 43204 Reus, Spain

**Keywords:** Viral infection, Health policy

## Abstract

Since the beginning of the COVID-19 pandemic and until September 2021, Spain suffered five waves of infection, the latter being related to the expansion of the Delta variant and with a high incidence. A vaccination campaign began in December 2020 and by the end of the fifth wave 77.3% of people had been fully vaccinated. Examining the changing dynamics of COVID-19 pandemic and its impact on outcomes among those hospitalized is essential. Our objective was to ascertain any differences in the characteristics and outcomes of hospitalized patients during that period compared to previous waves. We prospectively enrolled 200 consecutively admitted hospital patients from each wave and collected their clinical and demographic data from the medical records, including symptoms, comorbidities, deaths and whether they needed to be admitted to the Intensive Care Unit to receive assisted ventilation. We found that patients in the fifth wave were considerably younger than before, and the mortality rate fell from 22.5 to 2.0%. Admissions to the Intensive Care Unit decreased from 10 to 2%. Patients in the fifth wave had fewer comorbidities, and the age of the patients who died was higher than those who survived. Our results show a marked improvement in patient outcomes in the fifth wave, suggesting success of the vaccination campaign despite the explosion in cases due to the Delta variant.

## Introduction

Coronavirus disease-19 (COVID-19), produced by infection with severe acute respiratory syndrome coronavirus 2 (SARS-CoV-2) has so far infected an estimated 229 million people worldwide, causing 4.6 million deaths, according to figures reported by the WHO in September 2021^1^. Since the beginning of the pandemic until autumn 2021, Spain suffered five periods, or waves, of infection. The Delta variant was of particular concern as its transmission and viral load were considerably higher than those of the other variants detected so far and is associated with a lower neutralizing capacity of antibodies in vaccinated or convalescent people^2–8^. In the autonomous region of Catalonia, Spain, the Alpha variant (B.1.1.7) was the predominant one during most of the pandemic, although a significant number of cases of Beta (B.1.351) and Gamma (P.1) variants were also reported. However, as of April 16, 2021, the incidence of the Delta variant (B.1.617.2) increased exponentially, displacing the other variants and being responsible for around 90% of infections by mid-June^9^.

People at highest risk of hospitalization and death for COVID-19 are those of advanced age and those with age-associated diseases, such as type 2 diabetes mellitus, cardiovascular and respiratory diseases, and cancer. For this reason, vaccination campaigns have been aimed especially at people over 60 years of age, without neglecting the rest of the population, and have achieved a significant reduction in mortality. The COVID-19 vaccination campaign in Spain began on December 27, 2020. By the start of the fifth wave, when the Delta variant began to predominate, the number of people fully vaccinated was around 30% of the population, and at the time of our data analysis, in October 2021, it was 77.3%. The vaccines administered in our country are Comirnaty (Pfizer/BioNTech), Vaxzevria (Astra-Zeneca), Spikevax (Moderna) and Jcovden (Janssen). The emergence of new variants and the success of the vaccination campaign are recent features of the pandemic that are pulling in opposite directions, so the outcome is hard to predict. Understanding the impact of these characteristics on the COVID-19 burden is essential if we are to fine-tune the vaccination strategy on a global scale and be prepared for the appearance of new potentially aggressive variants.

The objective of the present study was to ascertain if there were differences in the characteristics and outcomes of the fifth wave patients admitted to our center compared to those of the previous waves.

## Methods

### Study design and participants

We conducted a prospective study on patients who had attended the *Hospital Universitari de Sant Joan* during the first, second, third and fifth waves of the pandemic, between March 14, 2020, and September 15, 2021. The first wave began in March 2020 and lasted through to June 21, 2020, during which a strict curfew and mandatory home isolation of the entire population was implemented, with the exception only of essential activities. The second wave then ran from June 22 to December 6, 2020, which coincided with a partial relaxation of restrictive measures. The third wave then spanned from December 7, 2020, to March 14, 2021, which was linked to Christmas and New Year holidays and the subsequent gatherings of families and friends. The fourth wave, from March 15 to June 19, 2021, was more like a "rebound" of the third, less intense, and with relatively few seriously ill patients, probably since a significant percentage of the older population had by that time been vaccinated. Finally, the fifth wave ran from June 20, 2021, to September 15, 2021, linked to the appearance of the Delta variant, which had a very high incidence^10^.

Ours is a reference hospital with 352 beds located in Reus, a medium-sized city (100,000 inhabitants) that offers health coverage to the surrounding, basically rural, counties, comprising a total of about 250,000 inhabitants, and including primary care centers and residences for the elderly. As per protocol, all patients who attended the hospital for admission or were treated in the Emergency Department underwent a reverse transcription polymerase chain reaction (RT-PCR) for SARS-CoV-2 with the aim of isolating positive cases to avoid nosocomial infection, regardless the reason for admission or if they had symptoms or not. The only exception to this rule was that during the first two weeks of the pandemic, RT-PCR analyses were only performed in those patients with suspected SARS-CoV-2 infection because our hospital did not have the test ready yet and the samples had to be sent to the *Hospital Clínic* in Barcelona. At the end of March 2020, our laboratory was already able to carry out the RT-PCR tests with a high-throughput system and since then and until the end of the study, they have been done in all admitted patients.

We prospectively included in the study the first 200 consecutive patients from each wave. The only inclusion criterion was being a hospitalized patient with a positive SARS-CoV-2 laboratory diagnosis. The exclusion criteria were: (1) Being a patient admitted with suspected SARS-CoV-2 infection but without RT-PCR confirmation or with a positive RT-PCR outside the study interval. (2) Being an outpatient with a laboratory diagnosis of SARS-CoV-2 infection. Therefore, our study was conducted in hospitalized patients with COVID-19 and admitted for having symptoms of COVID-19 or for other reasons. RT-PCR tests were carried out using the Procleix^®^ method in a Panther automated extractor and amplifier (Grifols Laboratories, Barcelona, Spain) or the VIASURE *SARS*-*CoV-2* Real Time PCR Detection Kit (CerTest Biotec, Zaragoza, Spain). All experimental protocols were approved by the *Comitè d’Ètica i Investigació en Medicaments* (Institutional Review Board) of *Hospital Universitari de Sant Joan* (Resolution CEIM 040/2018, amended on 16 April 2020), and methods were carried out in accordance with relevant guidelines and regulations. Informed consent was obtained from all participants in the study.

### Collection of clinical and demographic variables

The clinical and demographic data of the patients were collected from the medical records by the research staff, after receiving consent of the patients or their closest relatives. Consent was given orally to avoid contact with sheets of paper that could be a source of contagion. None of the patients or their relatives refused.

### Calculation of sample size

Accepting an alpha risk of 0.05 and a beta risk of less than 0.2 in a bilateral contrast, 195 subjects in each group were needed to register a difference greater than 20 cases in the variable “number of deaths”. A follow-up loss rate of 0% had been estimated. The ARCSINE approach was used. This mathematical function is commonly used to normalize data and homogenize variances when dealing with proportions or percentages^11^. We employed the GRANMO sample size calculator^12^. We do not present results from the fourth wave, since the number of hospitalized patients was too low (n = 135) to obtain reliable statistical results.

### Statistical analyses

Data is given as numbers and percentages or means and standard errors. Statistical comparisons between two groups were made using the χ^2^ test (categorical variables) or Student’s *t* test (quantitative variables). Statistical significance was set at P ≤ 0.05. All calculations were made using the SPSS 25.0 statistical package (SPSS Inc., Chicago, IL, USA).

## Results

The raw data is given as Supplementary Information (S1_File). Table 1 compares the values of the demographic and clinical variables in the patients of each of the waves. Patients from waves 1 to 3 had an advanced age (more than 60 years-old on average) together with age-related comorbidities, such as type 2 diabetes mellitus, hypertension, and cardiovascular diseases. In contrast, the age of patients from wave 5 was considerably lower, as was the percentage of comorbidities.

**Table 1:** Demographic and clinical characteristics of the first 200 patients hospitalized with COVID-19 belonging to each wave and studied at the *Hospital Universitari de Sant Joan de Reus*, between March 14, 2020 and September 15, 2021.

Variable	First wave (n = 200)	Second wave (n = 200)	Third wave (n = 200)	Fifth wave (n = 200)
**Demographic variables**				
Sex, female	90 (45.0)	92 (46.0)	92 (46.0)	100 (50.0)
Age	66.7 (1.3)	63.2 (1.4)	65.0 (1.5)	38.2 (1.6)^c,f,i^
Smokers	9 (4.5)	19 (9.5)^a^	19 (9.5)^a^	13 (6.5)
Alcohol drinking habit	9 (4.5)	14 (7.0)	17 (8.5)	16 (8.0)
**Department of admittance and outcome**				
Internal medicine	74 (37.0)	117 (58.5)^c^	94 (47.0)^a,d^	61 (30.5)^a,f,i^
Emergency unit	64 (32.0)	26 (13.0)^c^	45 (22.5)^a,e^	119 (59.5)^c,f,i^
Intermediate care unit	42 (21.0)	30 (15.0)	36 (18.0)	8 (4.0)^c,f,i^
Intensive care unit	12 (6.0)	19 (9.5)	16 (8.0)	4 (2.0)^e,h^
Gynecology	5 (2.5)	4 (2.0)	2 (1.0)	1 (0.5)
Pediatrics	3 (1.5)	2 (1.0)	6 (3.0)	3 (1.5)
Oncology	0 (0.0)	2 (1.0)	1 (0.5)	2 (1.0)
Days of admission	22.6 (1.8)	15.1 (1.3)^b^	12.4 (1.3)^c^	5.4 (0.4)^c,f,i^
COVID-19-related death^†^	45 (22.5)	27 (13.5)^a^	28 (14.0)^a^	4 (2.0)^c,f,i^
**Symptoms**				
Fever	131 (65.5)	132 (66.0)	97 (48.5)^c,f^	110 (55.0)^a,d,h^
Respiratory distress	118 (59.0)	112 (56.0)	124 (62.0)	55 (27.5)^c,f,i^
Pneumonia	116 (58.0)	117 (58.5)	118 (59.0)	49 (24.5) ^c,f,i^
Cough	101 (50.5)	85 (42.5)	66 (33.0)^c,d^	91 (45.5)
Diarrhea	44 (22.0)	39 (19.5)	22 (11.0)^b,d^	19 (9.5)^c,e^
Chills	42 (21.0)	7 (3.5)^c^	6 (3.0)^c^	5 (2.5)^c^
Acute kidney failure	21 (10.5)	37 (18.5)^b^	37^e^ (18.5)	4 (2.0)^c,f,h^
Acute respiratory distress syndrome	10 (5.0)	17 (8.5)	12 (6.0)	6 (3.0)^d^
Vomiting	9 (4.5)	29 (14.5)^c^	8 (4.0)^f^	13 (6.5)^e^
Other symptoms^‡^	11 (5.5)	55 (27.5)^c^	41 (20.5)^c^	42 (21.0)^c^
Asymptomatic	35 (17.5)	34 (17.0)	48 (24.0)^d^	25 (12.5)^h^
**Comorbidities**				
Cardiovascular disease	105 (52.5)	116 (58.0)	133 (66.5)^b,d^	41 (20.5)^c,f,i^
Type 2 diabetes mellitus	56 (28.0)	49 (24.5)	16 (8.0)^c,f^	19 (9.5)^c,f^
Chronic neurological disease	42 (21.0)	16 (8.0)^c^	20 (10.0)^b^	14 (7.0)^c^
Chronic lung disease	30 (15.0)	41 (20.5)	52 (26.0)^b^	16 (8.0)^a,f,i^
Chronic kidney disease	30 (15.0)	24 (12.0)	27 (13.5)	7 (3.5) ^c,e,i^
Cancer	28 (14.0)	29 (14.5)	22 (11.0)	16 (8.0)^a^
Concomitant infectious disease	4 (2.0)	9 (4.5)	18^b^ (9.0)	17 (8.5)^b^
Chronic liver disease	3 (1.5)	12^b^ (6.0)	14^b^ (7.0)	6 (3.0)
Pregnancy	1 (0.5)	5 (2.5)	1 (0.5)	4 (2.0)^d^
Postpartum	2 (1.0)	8 (4.0)	0^e^ (0.0)	2 (1.0)
**Respiratory intervention**				
Conventional oxygen therapy	151 (75.5)	130^a^ (65.0)	123^b^ (61.5)	42 (21.0)^c,f,i^
Invasive mechanical ventilation	27 (13.5)	11^b^ (5.5)	14^a^ (7.0)	6 (3.0)^c^
High-flow oxygen therapy	18 (9.0)	28 (14.0)	11^e^ (5.5)	11 (5.5)^e^
Non-invasive mechanical ventilation	7 (3.5)	25^b^ (12.5)	10^e^ (5.0)	3 (1.5)^f,g^

The total number of patients admitted to hospital for COVID-19 was 1283 and the total number of deaths was 131 (First wave: 204 patients and 49 deaths, 24.0%; Second: 264 and 35, 13.2%; Third: 305 and 22, 7.2%; Fourth: 135 and 6, 4.4%; Fifth: 375 and 19, 5.0%). For this study, the first 200 of each of the waves were prospectively selected, except for the fourth wave, which was discarded because it did not reach the minimum sample size. ^†^Any death in a patient with positive RT-PCR was recorded as COVID-19-related death. ^‡^Anosmia, ageusia, odynophagia, headache, anorexia, hyporexia, myalgia, and arthralgia. Results are given as numbers and percentages or as means and SEM. ^a^*P* < 0.05, ^b^*P* < 0.01, ^c^*P* < 0.001, with respect to the first wave; ^d^*P* < 0.05, ^e^*P* < 0.01, ^f^*P* < 0.001, with respect to the second wave; ^g^*P* < 0.05, ^h^*P* < 0.01, ^i^*P* < 0.001, with respect to the third wave.

**Figure 1:**
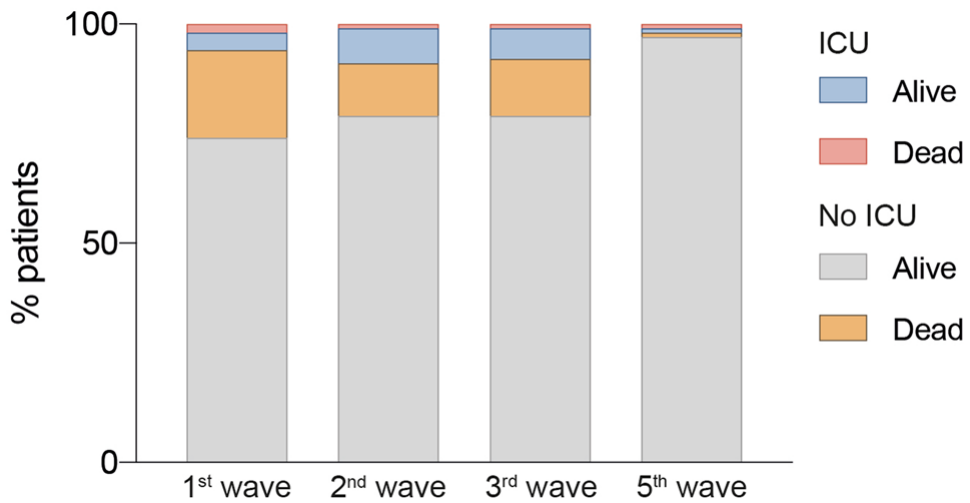
Percentage of patients admitted to the Intensive Care Unit (ICU) or not, in each of the waves studied.

Twenty-two point five percent of the hospitalized patients in the first wave died, and this percentage was reduced to 2.0% in the fifth wave. Likewise, while patients in the first three periods were admitted mostly to the Internal Medicine department, with average stays of more than 12 days, in the fifth they were treated mainly in the Emergency Department and the average stay reduced to 5.4 days. In addition, the percentage of patients moved to the Intensive Care Unit decreased significantly, from 6 to 10% in the first three waves to 2% in the fifth wave and the percentage of patients requiring assisted respiratory intervention also decreased (Fig. 1). The most common symptoms in all waves were fever, respiratory distress, and cough.

We next investigated the factors associated with the death of patients in the fifth wave, since these were generally younger and less severe than those in the other waves. Results should be treated with caution since only four patients died, making any statistical analysis rather weak. The patients who died were older than those who survived and were affected more commonly by age-related comorbidities, such as cardiovascular disease, type 2 diabetes mellitus, and chronic lung diseases (Table 2). Moreover, they were hospitalized for longer and more frequently needed assisted ventilation. It is noteworthy that these characteristics make these patients more like the deceased of waves 1–3 than to the survivors of wave 5 (Table 3).

**Table 2:** Differences between the clinical and demographic characteristics between the patients who survived and those who did not, belonging to the fifth wave of COVID-19 (from June 21, 2021 to September 15, 2021) studied at the *Hospital Universitari Sant Joan de Reus*.

Variable	Deceased		P-value
	No (n = 196)	Yes (n = 4)	
**Demographic variables**			
Sex, female	98 (50.0)	2 (50.0)	0.689
Age	37.7 (1.6)	66.0 (10.6)	0.012
Smokers	13 (6.6)	0 (0.0)	0.763
Alcohol drinking habit	15 (7.6)	1 (25.0)	0.285
**Symptoms**			
Fever	108 (55.1)	2 (50.0)	0.610
Respiratory distress	52 (26.5)	3 (75.0)	0.064
Pneumonia	48 (24.5)	2 (50.0)	0.495
Cough	90 (45.9)	1 (25.0)	0.381
Diarrhea	19 (9.7)	0 (0.0)	0.669
Chills	5 (2.6)	0 (0.0)	0.903
Acute kidney failure	4 (2.0)	0 (0.0)	0.922
Acute respiratory distress syndrome	5 (2.6)	1 (25.0)	0.116
Vomiting	13 (6.6)	0 (0.0)	0.763
Other symptoms^†^	42 (21.4)	0 (0.0)	0.386
Asymptomatic	24 (12.2)	1 (25.0)	0.416
Days of admission	2.1 (4.7)	18.7 (9.9)	< 0.001
**Comorbidities**			
Cardiovascular disease	38 (19.4)	3 (75.0)	0.028
Type 2 diabetes mellitus	17 (8.7)	2 (50.0)	0.046
Chronic neurological disease	13 (6.6)	1 (25.0)	0.254
Chronic lung disease	13 (6.6)	3 (75.0)	0.002
Chronic kidney disease	7 (3.6)	0 (0.0)	0.866
Cancer	15 (7.6)	1 (25.0)	0.285
Concomitant infectious disease	17 (8.7)	0 (0.0)	0.699
Chronic liver disease	6 (3.1)	0 (0.0)	0.884
Pregnancy	4 (2.0)	0 (0.0)	0.922
Postpartum	2 (1.0)	0 (0.0)	0.960
**Respiratory intervention**			
Conventional oxygen therapy	39 (19.9)	3 (75.0)	0.030
Invasive mechanical ventilation	5 (2.6)	1 (25.0)	0.116
High-flow oxygen therapy	11 (5.6)	0 (0.0)	0.796
Non-invasive mechanical ventilation	2 (1.0)	1 (25.0)	0.059

The total number of patients admitted to hospital in the fifth wave was 375, of which 19 died (5.0%). The first 200 patients were selected prospectively for this study. ^†^Anosmia, ageusia, odynophagia, headache, anorexia, hyporexia, myalgia, and arthralgia. Results are given as numbers and percentages or as means and standard errors.

**Table 3:** Differences between the clinical and demographic characteristics between the patients who survived and those who did not, belonging to waves 1–3 of COVID-19 (from March 15, 2020 to March 14, 2021) studied at the *Hospital Universitari Sant Joan de Reus*.

Variable	Deceased		*P*-value
	No (n = 500)	Yes (n = 100)	
**Demographic variables**			
Sex, female	231 (46.2)	43 (43.0)	0.558
Age	63.3 (0.9)	72.9 (18.9)	< 0.001
Smokers	42 (8.4)	5 (5.0)	0.675
Alcohol drinking habit	31 (6.2)	9 (9.0)	0.759
**Symptoms**			
Fever	301 (60.2)	57 (57.0)	0.626
Respiratory distress	291 (58.2)	61 (61.0)	0.655
Pneumonia	295 (59.0)	61 (61.0)	0.691
Cough	210 (42.0)	40 (40.0)	0.402
Diarrhea	96 (19.2)	9 (9.0)	0.089
Chills	45 (9.0)	9 (9.0)	0.018
Acute kidney failure	57 (11.4)	17 (17.0)	0.447
Acute respiratory distress syndrome	24 (4.8)	15 (15.0)	0.002
Vomiting	43 (8.6)	3 (3.0)	0.272
Other symptoms^†^	85 (17.0)	22 (22.0)	0.611
Asymptomatic	96 (19.2)	21 (21.0)	0.886
Days of admission	16.9 (1.0)	15.5 (1.7)	0.564
**Comorbidities**			
Cardiovascular disease	281 (56.2)	73 (73.0)	0.006
Type 2 diabetes mellitus	86 (17.2)	35 (35.0)	0.001
Chronic neurological disease	60 (12.0)	18 (18.0)	0.406
Chronic lung disease	100 (20.0)	23 (23.0)	0.813
Chronic kidney disease	55 (11.0)	25 (25.0)	< 0.001
Cancer	58 (11.6)	21 (21.0)	0.065
Concomitant infectious disease	25 (5.0)	6 (6.0)	0.965
Chronic liver disease	22 (4.4)	7 (7.0)	0.727
Pregnancy	6 (1.3)	0 (0.0)	0.963
Postpartum	9 (1.8)	1 (1.0)	0.947
**Respiratory intervention**			
Conventional oxygen therapy	320 (64.0)	82 (82.0)	0.005
Invasive mechanical ventilation	38 (7.6)	14 (14.0)	0.216
High-flow oxygen therapy	46 (9.2)	11 (11.0)	0.916
Non-invasive mechanical ventilation	33 (6.6)	9 (9.0)	0.832

The total number of patients admitted to hospital between the first and the third waves was 773, of which 106 died (13.7%). The first 200 patients were selected prospectively for this study. ^†^Anosmia, ageusia, odynophagia, headache, anorexia, hyporexia, myalgia, and arthralgia. Results are given as numbers and percentages or as means and standard errors.

All patients admitted during the first three waves and most of the patients admitted during the fifth wave were not vaccinated (Table 4). One hundred and forty-two surviving patients (71.0%) had not received any dose of vaccine and 58 (29.0%) had received at least one dose. During the study period only a maximum of two doses of vaccine had been administered, since the third booster dose campaign had not yet been started. However, the four licensed vaccines currently available in Spain produced a high protective immune response in people vaccinated with two doses (Comirnaty, Spikevax and Vaxzevria) or with one dose (Jcovden)^13^.

**Table 4:** Number of patients vaccinated, and vaccine administered.

Variable	First wave (n = 200)	Second wave (n = 200)	Third wave (n = 200)	Fifth wave (n = 200)
Vaccinated	–	–	–	58 (29.0)
Full schedule	–	–	–	28 (14.0)
Partial schedule	–	–	–	30 (15.0)
**Vaccine**				
Comirnaty Pfizer-BioNTech	–	–	–	35 (17.5)
Vaxzevria (AstraZeneca)	–	–	–	11 (5.5)
Jcovden (Janssen)	–	–	–	7 (3.5)
Spikevax (Moderna)	–	–	–	5 (2.5)

Percentages are calculated with respect to the total number of cases (n = 200).

## Discussion

Our results for patients taken from waves 1 to 3 were like those reported previously by many studies around the world^13–17^. However, the appearance of new variants at the same time as the acceleration in the global vaccination campaign has created a new scenario, and at the moment seems to cause a relief for health systems^4,18,19^. Our experience with the fifth wave was very different from that of the previous ones, not only because the number of deaths was much lower, but also because the time occupied by hospital beds was considerably reduced. Indeed, most of the patients who came to the emergency room were young, with mild symptoms, and they had no indication of need for ICU care. Therefore, our hospitalization activity was not pressured, which helped to continue with medical and surgical activities (Table 1).

These results are an empirical verification of the predictions previously drawn up in mathematical models that concluded that vaccination would markedly reduce the risk of COVID-19 resurgence and adverse outcomes. This is also backed up by data from South Korea^20^, the United States^9^, Germany^21^, and The Netherlands^22^. In contrast to that, the explosion of the Delta variant was linked to a large increase in adverse events and deaths in India, where the vaccination campaign was delayed by enormous logistical difficulties^23^.

It is well known that the efficacy of vaccines depends largely on whether the full or only partial regimen has been administered, or indeed the time elapsed from vaccination to infection with SARS-CoV-2. For example, a study in Canada has reported that vaccine effectiveness against Delta after partial vaccination was lower compared to Alpha with both the Moderna (72% vs. 83%) and the Pfizer (56% vs. 66%) vaccines, while full vaccination increased protection to comparable levels (87%)^24^. In addition, a British study reported that effectiveness was lower against Delta after one dose of Pfizer or Astra-Zeneca vaccines compared to Alpha (30.7% vs. 48.7%), and increased after the second dose, although it was always lower for the Delta variant^25^. A prospective cohort study in our same geographic area showed that vaccination was associated with 80–91% reduction in SARS-CoV-2 infection in nursing home residents, nursing home staff, and healthcare workers and reductions in hospital admissions and mortality among nursing home residents for up to 5 months^26^. Likewise, a complete vaccination schedule reduced SARS-CoV-2 infection by 81.2% in people over 65 years of age admitted to nursing homes^27^.

However, the short time that SARS-CoV-2 has been associated with humans means that the duration of immune memory and protective immunity after COVID-19 and in response to COVID-19 vaccines is still unknown^28^. Although the effectiveness of COVID-19 vaccines is still very high in older people, it must be considered that their immune response may be lower. In the elderly, as in the rest of the population, other factors also influence the effectiveness of the vaccine, such as the previous immune status, the type of vaccine or the time elapsed since vaccination^29^.

In conclusion, our results show a marked decrease in the severity of disease of infected patients, in the number of admissions to the Intensive Care Unit, and in the number of deaths, too, in hospitalized patients during the fifth wave of the pandemic in Spain. This was linked to the relative youth of the patients and the absence of comorbidites. This improvement coincided with most of the population, and especially all the elderly, having been vaccinated, despite the explosion of the Delta variant.

Our study has several limitations. First, not all patients admitted with each wave were included in the study, but only the first 200, and the study was performed in patients with a COVID-19 positive RT-PCR, independently on the reason for admission. They were, then, hospitalized patients with COVID-19, not for COVID-19. In addition, surgical or traumatological patients were not included since non-urgent operations in positive COVID-19 patients were postponed until their RT-PCR was negative.

Moreover, the criteria for performing RT-PCR tests changed from the end of March 2020. The increase in the number of tests since then likely identified many more asymptomatic cases which were missed in the first weeks of the epidemic when only symptomatic people were tested. In addition, during the first wave, many patients presented to the hospital with severe respiratory failure and needed to be transferred to the Intensive Care Unit immediately to receive orotracheal intubation. In the subsequent waves, the patients went to their family doctor earlier, so treatment began earlier, the need for admission to Intensive Care was not as frequent, and mortality decreased. For example, in the fifth wave, there was a significant increase in patients seen in the Emergency Department, who were sent home the same day or 24 h later and who did not require admission to the hospitalization ward. The groups, therefore, are not totally homogeneous. Finally, this was a single-center study in a specific region of Mediterranean Europe.

Despite these limitations, we think that the results can be extrapolated to regions and countries that have had similar circumstances, i.e., a high incidence of the Delta variant together with a high vaccination rate. At a time when the elderly population is already largely protected, a special effort must be made to vaccinate young people to achieve group immunity as soon as possible.

## Supplementary Information

Supplementary Information.

## Data Availability

Supporting datasets are available as Supplementary Information (S1_File.xlsx).
